# The *Non-Coding RNA* Journal Club: Highlights on Recent Papers—11

**DOI:** 10.3390/ncrna8030031

**Published:** 2022-05-05

**Authors:** Hélène Bonnet, Baptiste Bogard, Florent Hubé, Mirolyuba Ilieva, Shziuka Uchida, Maria Ascensión Ariza-Mateos, Alexander Serganov, Barbara Pardini, Alessio Naccarati, Gaetano Santulli, Fahimeh Varzideh, Hua Xiao, Patrick K. T. Shiu

**Affiliations:** 1 UMR7216 Épigénétique et Destin Cellulaire, CNRS, Université de Paris, F-75013 Paris, France; 2 Center for RNA Medicine, Department of Clinical Medicine, Aalborg University, DK-2450 Copenhagen SV, Denmark; mirolyubasi@dcm.aau.dk; 3 Department of Biochemistry and Molecular Pharmacology, New York University Grossman School of Medicine, New York, NY 10016, USA; maria.arizamateos@nyulangone.org; 4 Candiolo Cancer Institute-FPO IRCCS, 10060 Candiolo, Italy; 5 Italian Institute for Genomic Medicine (IIGM), IRCCS Candiolo, 10060 Candiolo, Italy; 6 Albert Einstein College of Medicine, New York, NY 10461, USA; fahimeh.varzideh@einsteinmed.edu; 7 Division of Biological Sciences, University of Missouri, Columbia, MO 65211, USA; xiaohu@missouri.edu

## 1. Introduction

We are delighted to share with you our eleventh Journal Club and highlight some of the most interesting papers published recently. We hope to keep you up-to-date with non-coding RNA research that is outside your study area. The *Non-Coding RNA* Scientific Board wishes you an exciting and fruitful read.

## 2. A Novel Strategy to Deliver Exogenous DNA Using Bacterial Retroelements

### Highlight by Hélène Bonnet and Florent Hubé

Exogenous DNA is used as a template to alter gene expression and edit or insert modifications in genomes, but there are issues with its delivery, abundance, targeting, and efficiency. Small bacterial retroelements called retrons have gained attention as intracellularly generated DNA eliminates some of these obstacles. They contain a ncRNA sequence that includes a retrotranscribed part (RT-DNA) and a reverse transcriptase coding sequence. Their size and well-defined regulatory sites, along with their lack of host factor requirements, make retrons a great strategic choice for genome editing across kingdoms.

In this study, the authors (Lopez et al.) extended the a1/a2 ends of the ncRNA sequence in Eco1 and Eco2 retrons from *E. coli*, which increases RT-DNA production in bacteria and yeast [1]. Moreover, after modifying part of the ncRNA sequence to produce the desired RT-DNA template, the genome editing rate and preciseness are positively correlated with increased RT-DNA abundance. Finally, when the retrons are transfected in combination with Cas9 and a guide RNA, precise editing increases in eukaryotic cells.

Thus, novel sequence editing of ncRNA in bacterial retrons allows for new genome modification strategies, with or without CRISPR-Cas support, using a universal architecture. Furthermore, the authors proved that an abundance of RT-DNA is a limiting factor for genome editing efficiency.

## 3. H/ACA snoRNA Acrobatics: A Novel Strategy to Direct Synthesis of Adjacent Pseudourines

### Highlight by Baptiste Bogard and Florent Hubé

The isomerization of uridines into pseudouridines (Ψs), termed pseudouridylation, is one of the most widespread RNA modifications found in rRNAs, snRNAs, and tRNAs. RNA pseudouridylation in snRNAs and rRNAs is guided by H/ACA small nucleolar RNAs (snoRNAs) associated with the Ψ synthase dyskerin. H/ACA snoRNAs consist of two hairpins composed of an inner loop that hybridizes with the target RNA to direct the pseudouridylation. Consequently, one pseudouridylation loop guides only one Ψ.

In the paper by Jády et al. [2], they showed that two adjacent Ψs (ΨΨ or ΨNΨ) can be synthesized by the same pseudouridylation loop of a H/ACA snoRNA. For instance, SNORA53, which is predicted to direct the synthesis of Ψ3747 and Ψ3749 in the human 28S rRNA, adopts two different structural conformations at the level of its loop and thus provides guidance for both pseudouridylations. The authors also found three other H/ACA snoRNAs with dual guidance activity and discovered that these strategies were evolutionarily conserved.

In summary, H/ACA guides RNA acrobatics, as defined by the authors, representing a novel, unexpected, and conserved strategy of snoRNAs for directing the synthesis of adjacent Ψs in heavily-pseudouridylated sequences, contributing to the synthesis of at least 6% of Ψs in snRNAs and rRNAs.

## 4. Paving the Way for lncRNA Drugs: Benefit of Exercise to Heart Functions

### Highlighted by Mirolyuba Ilieva and Shziuka Uchida

It is a well-known fact that lncRNAs are much less species-conserved than protein-coding genes. Yet, some lncRNAs are conserved across species, which suggests the importance of these lncRNAs in the development and physiopathology of organisms. To demonstrate the functional importance of human lncRNAs, the usage of model animals is of utmost importance as it is difficult to recapitulate the in vivo situations in cultured human cells. Additionally, manipulating lncRNAs in vivo is necessary for drug development.

In the paper by Li et al. [3], they identified a species-conserved lncRNA, *lncExACTs*, which is downregulated by physical exercise and upregulated in mouse models of cardiac hypertrophy and heart failure patients. Mechanistically, *lncExACT1* binds *miR-222* and modulates Hippo/Yap signaling via acting in *cis* to regulate the antisense overlapping protein-coding gene, *DCHS2*. By injecting gapmer against *lncExACT1* in the tail vein of mice, similar phenotypes to those discovered after 8 weeks of exercise were observed, as well as increased protection against cardiac dysfunction and fibrosis in a mouse model of pathological cardiac hypertrophy.

In summary, this study functionally and mechanistically characterized a species-conserved lncRNA using model organisms and shed light on the potential development of interventions targeting lncRNA using antisense oligos, which are approved by the FDA, with examples including nusinersen (Spinraza).

## 5. Riboswitches: Loaded with a Double Charge

### Highlight by Maria Ascensión Ariza-Mateos and Alexander Serganov

Riboswitches are non-coding RNA elements typically located upstream from important bacterial genes that are capable of modulating gene expression via binding to small molecule effectors. The vast majority of riboswitches specifically recognize a single cellular metabolite. Some riboswitches contain two or more metabolite-sensing domains arranged in tandem and controlled by their own effectors. Occasionally, a single domain recognizes more than one small molecule.

Schroeder et al. [4] recently showed that the regulation of cellular concentrations of queuosine (Q)—a hypermodified 7-deazapurine nucleobase required for translational fidelity—involves a riboswitch that binds two molecules of the precursor preQ1 in *Carnobacterium antarcticus*. Previously identified preQ1 riboswitches bind a single molecule of the effector and, given that the preQ1 riboswitch is one of the smallest riboswitches, this new finding has surprised the research community. The X-ray crystal structure revealed that the riboswitch accommodates two stacked preQ1 molecules in a single binding pocket. Biochemical and microbiological experiments revealed positive cooperativity in ligand binding and suggested the importance of double ligand binding for a response at low concentrations of the ligand. Remarkably, the double-preQ1-binding riboswitches have been confirmed in a few other bacteria and are possibly present in more species.

## 6. Specific Motifs in the microRNA Sequence Drive Their Secretion in Small Extracellular Vesicle or Retention in the Cell of Origin in a Cell-Type-Specific Manner

### Highlight by Barbara Pardini and Alessio Naccarati

The bidirectional crosstalk between cells and their microenvironment is a crucial element under both normal physiology and pathological conditions. Together with cell–cell contact or the secretion of soluble molecules (such as cytokines or growth factors), the trafficking of small extracellular vesicles (sEVs) has emerged in the last ten years as a new promising mode of cell-to-cell communication. Exosomes and other sEVs may contain different cargos, including various types of non-coding RNAs, that are transferred from a donor to a recipient cell. This represents a form of inter-cellular communication, leading to changes in gene expression and cellular function both in health and disease states. However, how the non-coding RNAs are sorted as sEVs or retained in cells remains a mechanism that is largely unexplored.

Garcia-Martin and colleagues recently contributed to elucidating the complex regulation of the selection of molecules to be transported in sEVs [5]. The authors demonstrated that microRNAs (miRNAs) contain specific sorting sequences (called EXOmotifs and CELLmotifs by the authors) that address these molecules to their secretion in sEVs or retention in the cells of origin. Although this was partially already known [6], Garcia-Martin et al. found that different cell types, including white and brown adipocytes, endothelium cells, liver cells, and muscle cells, make preferential use of specific sorting sequences that define the sEV miRNA profile of that cell type. Data indicate that miRNA sorting into sEVs is a complex, integrated system involving multiple motifs that contribute to sorting and cellular retention in a cell-type-specific manner. Moreover, the authors found that Alyref and Fus, two RNA-binding proteins, are responsible for recognizing and loading miRNAs carrying one of the strongest EXOmotifs, CGGGAG, into sEVs.

Finally, the insertion/deletion of a specific motif in a miRNA decides its fate regarding whether it is retained in the cell of production or secreted into exosomes/sEVs, opening the possibility of regulating miRNA retention or secretion for therapeutic benefits. Defining the full miRNA sorting machinery involved in sEV secretion will help better relate circulating miRNAs to their tissue of origin and improve our understanding of the circulation of miRNAs in diseases.

## 7. miR-210 Is a Master Regulator of Cardiac Mitochondrial Bioenergetics

### Highlight by Gaetano Santulli and Fahimeh Varzideh

Ischemic heart disease remains the leading cause of death worldwide. Thus, therapeutic strategies aiming at attenuating the detrimental effects of acute myocardial infarction are dramatically needed. Although mitochondrial dysfunction is known to play a crucial role in ischemic heart disease, mechanistic therapeutic approaches targeting mitochondria are lacking. In an elegant paper recently published in *Circulation* [7], Lubo Zhang and collaborators convincingly demonstrated that miR-210 controls mitochondrial bioenergetics and the generation of reactive oxygen species in a murine model of myocardial infarction. The investigators identify and validate mitochondrial glycerol-3-phosphate dehydrogenase (GPD2) as a highly conserved target of miR-210. GPD2 is a protein bound to the outer face of the mitochondrial inner membrane that, alongside the cytosolic GPD1, orchestrates the glycerophosphate shuttle between mitochondrial and cytosolic energy production and redox balance. Indeed, GPD2 produces ~40–50% of the total H_2_O_2_-generating capacity in murine heart mitochondria. 

By combining in vivo and in vitro experiments, the authors showed that miR-210 promotes the mitochondrial metabolic switch from oxidative phosphorylation to glycolysis, thereby fine-tuning cardiac responses to myocardial infarction, with major implications in the fields of cardiovascular medicine and non-coding RNAs. Intriguingly, a robust phenotype was observed in male but not female mice, suggesting that miR-210 could be functionally involved in the regulation of the intricate interplay of sex-biased miRNA networks and intrinsic sex differences in mitochondrial genotype/phenotype, at least in cardiovascular disease.

## 8. Fungal Counterattack against Transkingdom RNA Interference

### Highlight by Hua Xiao and Patrick K. T. Shiu

During some plant–pathogen interactions, small RNAs are bidirectionally transmitted to silence targets in the recipient organisms in a phenomenon called transkingdom RNA interference (RNAi). For example, plants can deliver microRNAs (miRNAs) to fungal cells to suppress the expression of virulence genes. In a recent issue of *PNAS*, Chen Zhu and colleagues showed that fungi can antagonize this process by inhibiting the host’s miRNA export system [8].

Using culture filtrates of the phytopathogen *Verticillium dahliae*, Zhu et al. conducted mass spectrometry to look for secreted fungal effectors that could counteract transkingdom RNAi initiated by plants. Because miRNAs are mainly processed in the nucleus, the authors concentrated on candidates that could translocate to the plant nucleus (and act as a silencing suppressor). One such effector, named VdSSR1 (secretory silencing repressor 1), can do just that. VdSSR1 is essential for fungal virulence, as its removal greatly enhances plant-to-fungus miRNA trafficking and the transkingdom silencing of virulence genes. Mechanistically, VdSSR1 sequesters ALY adaptors of the TREX complex to inhibit the nuclear export of AGO1–miRNA complexes, leading to a reduction in cytoplasmic miRNAs (including those important for transkingdom RNAi).

This study demonstrated that the nuclear export of small RNAs can be exploited by a fungal pathogen to counteract plant immunity. Future investigations of this kind will reveal what other weapons and antagonistic mechanisms can be used in the arms race on the transkingdom RNAi front.
